# Tuberculosis of the wrist mimicking rheumatoid arthritis – A rare case

**DOI:** 10.1016/j.ijscr.2019.08.023

**Published:** 2019-09-05

**Authors:** Wildan Latief, Elfikri Asril

**Affiliations:** Department of Orthopaedic & Traumatology, Cipto Mangunkusumo National Central Hospital and Faculty of Medicine, Universitas Indonesia, Jalan Diponegoro No. 71, Jakarta Pusat, Jakarta 10430, Indonesia

**Keywords:** Wrist tuberculosis, Rheumatoid arthritis, Debridement, Tuberculous arthritis

## Abstract

**Introduction:**

*Mycobacterium tuberculosis* causes infection in approximately one-third of the world's population. Arthritis due to *Mycobacteriurn tuberculosis* usually presents as a chronic, slowly progressive, monoarticular infection that predominantly involves the weight-bearing joints and the spine. The hand and wrist are rare sites for tuberculosis (TB) and comprises of < 1% of all skeletal TB. Even though it is rare, TB of the wrist is a cause of great morbidity. We presented a case of wrist TB treated with debridement and synovectomy.

**Presentation of Case:**

A 35 years old woman came with the chief complaint of pain and swelling on the right wrist since 1 year ago and was diagnosed with rheumatoid arthritis of the wrist. Patient then was given methylprednisolone 4 mg twice a day and methotrexate 175 mg once a week. However, the pain and swelling of her right wrist became worse. Subsequently patient was admitted to hospital and laboratory findings suggested a tuberculous arthritis of the wrist. Debridement, synovectomy, and biopsy, in addition to antituberculosis therapy, were then performed.

**Discussion:**

Tuberculous arthritis is generally a monoarticular disease that typically involves the spine or large and medium-sized joints. Rheumatoid arthritis (RA) and tuberculous arthritis may have similar clinical characteristics, which consists of a chronic course with periarticular soft-tissue swelling.

**Conclusion:**

Tuberculous arthritis and RA can have similar characteristic but laboratory examination can help in establishing diagnosis.

## Highlights

• The involvement of the wrist in the tuberculous arthritis is very rare.
• Tuberculous arthritis of the wrist can cause great morbidity.
• Rheumatoid arthritis can mimick tuberculous arthritis.
• Tuberculous arthritis of the wrist can be successfully treated with debridement and synovectomy.

## 1 Introduction

*Mycobacterium tuberculosis* causes infection in approximately one-third of the world's population. Musculoskeletal infection by this bacteria is the most common form of extrapulmonary tuberculosis which accounts for 10–19% of the cases. Arthritis due to *Mycobacteriurn tuberculosis* usually presents as a chronic, slowly progressive, monoarticular infection that predominantly involves the weight-bearing joints and the spine. The joints and bursae of the forearm are less frequently affected [1].

The involvement of the wrist in the tuberculous arthritis is very rare, and the involvement of the wrist typically begins in the scapholunate joint [2]. The hand and wrist are rare sites for tuberculosis (TB) and comprises of < 1% of all skeletal TB. Even though it is rare, TB of the wrist is a cause of great morbidity. A frequent finding of the wrist TB is a delay in diagnosis causing residual stiffness and pain after treatment [3].

We presented a case of wrist TB treated with debridement and synovectomy. This work had been presented according to SCARE guideline [4].

## 2 Patient Information

A 35-year-old woman presented with wrist joint pain of 1 year. There was no skin rash, early-morning stiffness, or chronic cough. In the rheumatology polyclinic, she was diagnosed with rheumatoid arthritis of the right wrist. Patient then was given methylprednisolone 4 mg orally twice a day and methotrexate 175 mg once a week. One month after the therapy, the pain and swelling of her right wrist became worse and then patient was advised to be hospitalized for further evaluation and receiving intravenous antibiotic treatment.

## 3 Clinical findings

**Fig. 1:**
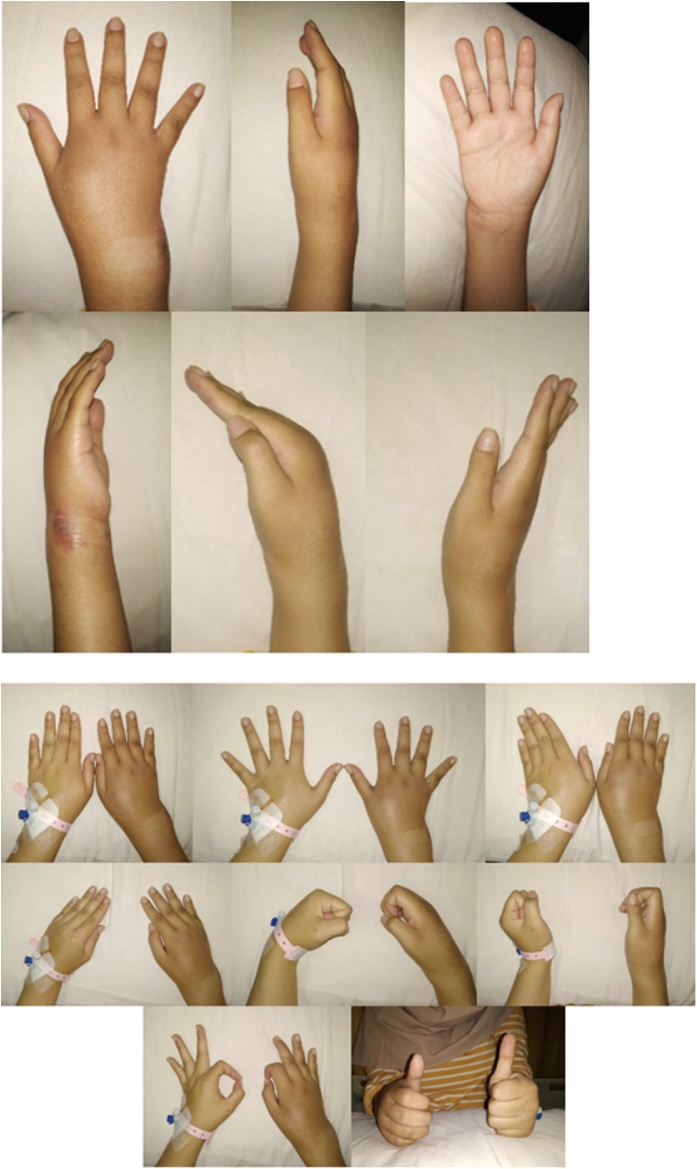
Clinical manifestation of the Patient.

On inspection, there were swelling on the right wrist with signs of inflammation including redness and warmth (Fig. 1). On palpation, tenderness with the VAS score of 2–3 on the right wrist was found. The range of motion of the wrist was also limited. Wrist flexion was 0-45°, wrist extension was 10° and ulnar and radial deviation was 10°. The ability to pinch was preserved in both hands but was decreased on the right wrist. The range of motion of the finger was limited due to pain.

## 4 Diagnostic Assessment

Laboratory examination of synovial fluid analysis and complete blood count were performed. Complete blood test showed an increased erythrocyte sedimentation rate (ESR) of 72 mm/h (normal 0–20 mm/h), negative rheumatoid factor (RF) (normal less than 40 U/mL), increased C reactive protein (CRP) of 10.4 mg/L (normal less than 3 mg/l), negative anticyclic citrullinated peptide antibody (ACPA), and leukocytosis. The cell count, PMN cell count, and mononuclear cell count were high, each with the value of 171,179 (normal 9100 – 28,700); 142,830 (normal 1900 – 11,500) and 22,940 cells/uL (normal 300 – 1400), respectively. Synovial fluid analysis showed that the fluid color was reddish with translucent-opaque clarity. The leukocyte count was 6–8 per field (normal less than 5). This concluded that the synovium contained amount of cells in line with septic arthritis.

**Fig. 2:**
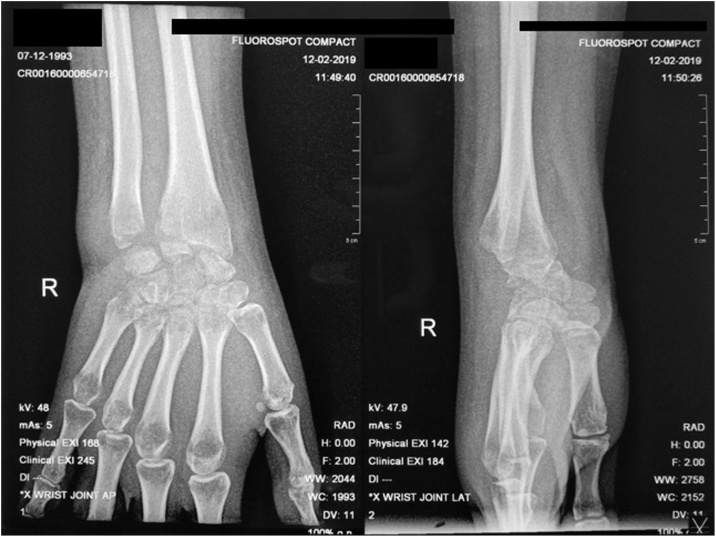
X-Ray Examination of the Wrist.

**Fig. 3:**
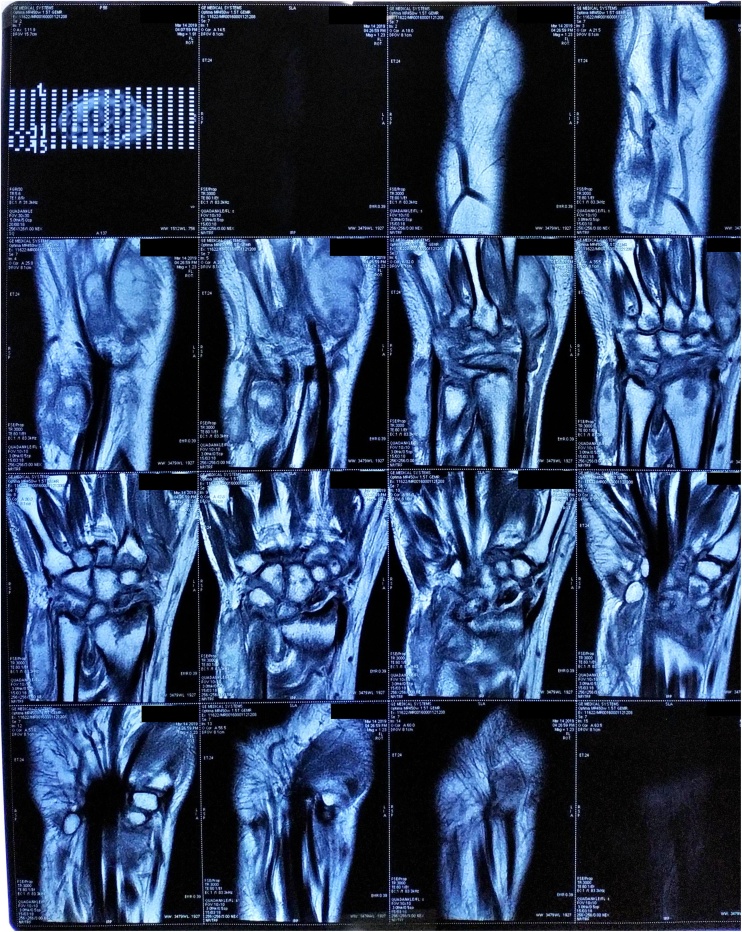
MRI of the wrist.

Power Doppler ultrasound (US) of the right wrist showed severe hyperemia. The x-ray finding was diffuse osteoporosis and irregularity of the radiocarpal surfaces, and dislocation of radiocarpal joint (Fig. 2). Whereas magnetic resonance imaging (MRI) finding was destruction of the radiocarpal joint, tenosynovitis changes of extensor carpi radialis brevis and extensor digitorum tendons with mild subcutaneous soft tissue swelling suggestive of cellulitis, with fluid in the joint (Fig. 3).

According to the new American College of Rheumatology (ACR)/ European League Against Rheumatism (EULAR) 2010 criteria for rheumatoid arthritis, the patient had score of 5 points, with 3, 1, 1, from joint, symptom duration, and acute-phase reactants, respectively. The patient did not fulfil the ACR/ EULAR criteria of at least 6 of 10 points. However, based on US and MRI findings, patient was treated orally with methotrexate 17.5 mg/week, and methylprednisolone 4 mg orally as bridge therapy. A month later, the symptoms had not improved. The joint pain persisted, and ESR remained high. Indeed, patient was dependent on celecoxib 90 mg for pain relief. US of the hands was repeated and showed similar findings. Aspiration of the swollen joint was attempted, and no crystals was found. Synovial biopsy was then considered to ascertain the underlying diagnosis, which showed caseating granulomatous inflammation suggestive of TB. In addition, it revealed that the synovium contained amount of cells in line with septic arthritis. The color of synovial fluid was reddish with translucent-opaque clarity. The leukocyte count was 6–8 per field. Acid-fast stain of the synovial fluid was negative for acid fast bacilli (AFB), but the culture grew *Mycobacterium tuberculosis*.

## 5 Theurapeutic intervention

**Fig. 4:**
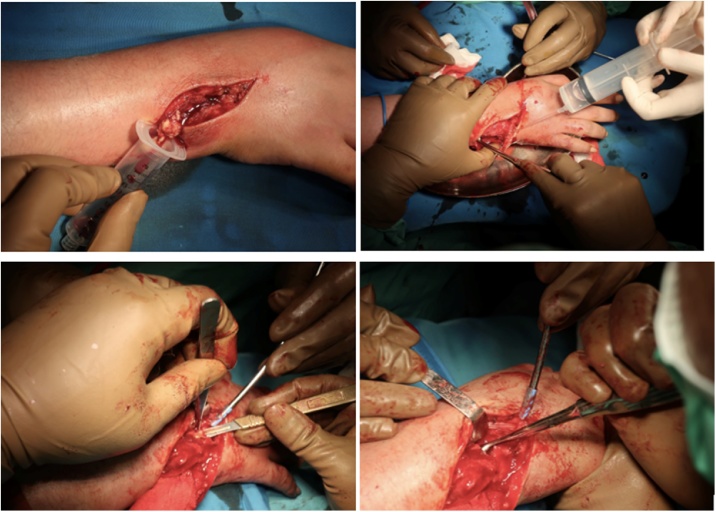
Intraoperative Procedures.

Initially before visiting us, patient was misdiagnosed to have rheumatic arthritis of the wrist and was given immunosuppressive drugs methylprednisolone and methotrexate. After further examination was performed in our center, patient was diagnosed with tuberculous inflammatory arthritis of the right wrist. The patient was started on 2 months of intensive therapy with isoniazid, rifampicin, ethambutol, and pyrazinamide then switched to 10 months of maintenance therapy with

isoniazid and rifampicin. Subsequently, patient was treated with debridement, synovectomy, and biopsy (Fig. 4).

## 6 Follow up and outcomes

**Fig. 5:**
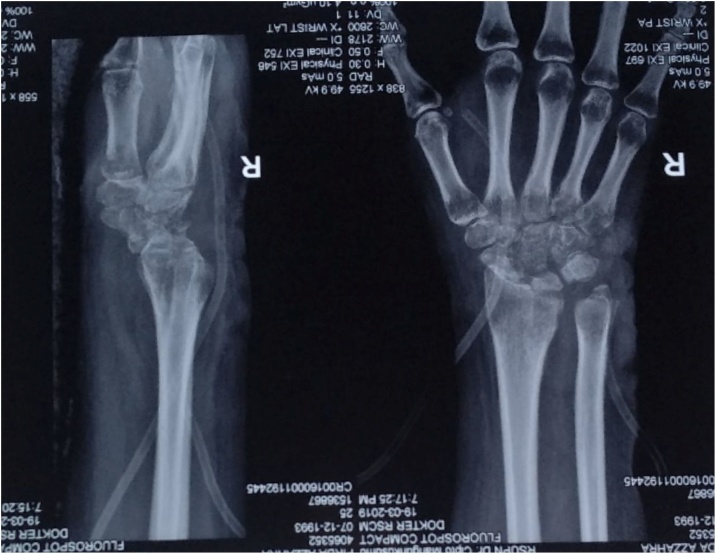
Postoperative X-Ray.

The sample of biopsy was brought to the histopathologic department. Postoperative x-ray was taken (Fig. 5).

## 7 Discussion

The incidence of TB has increased, even in the developed countries. The risk factors for increased incidence of TB are the absence of BCG vaccination, trauma or immunodeficiency (which can reactivate a pre-existing tubercular infection), and low socioeconomic status. This socioeconomic factor is the major factor. The musculoskeletal TB is in the fourth position after pulmonary, urogenital and ganglionic tuberculosis. After the shoulder, TB of the hand and wrist is the rarest musculoskeletal tuberculosis, which comprises 2–4% of all the localizations of the musculoskeletal system. Musculoskeletal tuberculosis is caused by hematogenous spread from an active or dormant pulmonary or gastrointestinal source. TB of the wrist slowly over several years, from an early stage where pain and swelling are the most common presenting features, until an advanced stage of articular destruction with abscess discharges [2]. Our case presented was a patient presented with tuberculous arthritis of the wrist that was first diagnoses as rheumatoid arthritis. This was a very rare case.

At an early stage, radiological signs only give little clue. The signs are diffuse osteoporosis and irregularity of the radiocarpal surfaces. The computed tomography (CT) scan examination is more specific for this disease. The geode and a joint space narrowing are the signs that strongly suggest the presence of the disease. The geode is usually in the scaphoid or the semi-lunar, without sequestration. Geode is a variable-sized joint space. The radiological signs undergo evolvement with the clinical picture. In the stage of articular destruction, there are multiple geodes, disappearance of articular space, and all the joints are affected including the trapezio-metacarpal joint. Moreover, all the bones are nibbled and deformed within a significant radiological blurring [2].

Tuberculosis in the wrist has a double etiopathogeny. The occurrence of tuberculous arthritis in the wrist is straightaway articular in two thirds of the cases, and it is secondary to a synovitis in one third of the cases, usually flexor tenosynovitis. The initial destruction begins in the external and proximal, then it progresses distally. The destruction will undergo evolvement until it comes to articular collapse, subluxation and deformation. Only a few abscesses present in these cases. When found, these abscesses are usually encapsulated by a solid barrier and synovial girdles that are difficult to penetrate. Secondary etiopathogenesis after synovitis occurs after several years of evolution [2].

Tuberculous arthritis is generally a monoarticular disease that typically involves the spine or large and medium-sized joints, such as the hip and knee. On the other side, rheumatoid arthritis (RA) is usually a symmetric polyarticular disease with commonly involves peripheral joints. However, RA may also manifest as a monoarticular disease and persist in such a picture for a long time. RA and tuberculous arthritis may have similar clinical characteristics, which consists of a chronic course with periarticular soft-tissue swelling. It also can have similar radiologic findings, such as periarticular osteoporosis, bone erosion, and presence of joint effusion, rendering differential diagnosis difficult. These reasons can cause the misdiagnosis of tuberculosis as rheumatoid arthritis [5].

The advantages of MRI are its superior soft-tissue contrast and its ability to depict changes in cartilage, ligaments, and synovial tissue, especially in the evaluation of early changes in tuberculous arthritis. MRI reveals heterogeneous low signal intensity in the joint because of rice bodies, cartilage fragments, and hemorrhage [5].

In our case, the patient first came with the chief complaint of pain on the wrist. Patient was misdiagnosed with rheumatoid arthritis and was given immunosuppressive drugs. This caused worsening of the condition because the true diagnosis was tuberculous arthritis. An early diagnosis would help achieve an almost complete cure with normal function. However, the main problem remains the difficulty in diagnosing osteoarticular TB due to the nonspecific clinical signs, which lead to multiple possible diagnoses. Differential diagnoses of tuberculous arthritis is RA, gouty arthritis, pigmented villonodular synovitis, and infection such as pyogenic or fungal infections

The treatment for this condition is easy, consisting of chemotherapy and orthopaedic treatments. However, only chemotherapy, consisting of anti-bacillary chemotherapy for 12 months of isoniazid, rifampicin, pyrazinamide and ethambutol, is essential. The orthopedic immobilization splint will be maintained until the disappearance of clinical signs (3–4 weeks), followed by rehabilitation. In surgical procedure, other than the biopsy, there is very little need for surgery.

Joint involvement occurs in 84% of the cases of the cases. The sites that are the most commonly affected are spine (50%), pelvis (12%), ribs (7%), hip and femur (10%,) shoulder and ankle (2%). The upper limbs are affected in only 4–10% of the cases of musculoskeletal tuberculosis. In developed countries tuberculous lesions of the elbow or wrist and the corresponding bursae are very rare, accounting for 2% of the cases of musculoskeletal tuberculosis [1].

## 8 Conclusion

This case report showed that the diagnosis of tuberculous arthritis was challenging because some conditions, including rheumatoid arthritis, can mimick the clinical appearance of tuberculous arthritis. This highlights the importance of considering the differential diagnosis of arthritis, in order to perform the proper examinations to establish the right diagnosis of the patient so that the given treatment does not worsen the patient’s condition or adding the morbidity to the patient.

## Sources of funding

The authors received no financial support for the research, authorship, and/or publication of this article.

## Ethical approval

The ethical approval was not required for this case report.

## Consent

Informed consent had been obtained from the patient before the manuscript was written.

## Author contribution

Wildan Latief: study concept, data collection, data interpretation, and writing the paper.

Elfikri Asril: data collection, data interpretation and writing the paper.

## Registration of research studies

None.

## Guarantor

Wildan Latief.

## Provenance and peer review

Not commissioned, externally peer-reviewed.

## Declaration of Competing Interest

The authors certify that they have No affiliations with or involvement in any organization or entity with any financial interest or non-financial interest in the subject matter or materials discussed in this manuscript.

## References

[1] Hunfeld K.P., Rittmeister M., Wichelhaus T.A., Brade V., Enzensberger R. (1998). Two Cases of Chronic Arthritis of the Forearm Due to Mycobacterium tuberculosis.

[2] Ali M., Benzarti S. (2015). Case Report A rare localization of tuberculosis of the wrist: the scapholunate joint 5. Int. J. Mycobacteriol..

[3] Shah M.A., Shah I. (2018). Case Report Wrist Swelling – Is It Tuberculosis?.

[4] Agha R.A., Borrelli M.R., Farwana R., Koshy K., Fowler A., Orgill D.P., For the SCARE Group (2018). The SCARE 2018 statement: updating consensus surgical CAse REport (SCARE) guidelines. Int. J. Surg..

[5] Choi J., Kang H.S. (2009). Rheumatoid Arthritis and Tuberculous Arthritis: Differentiating MRI Features.

